# Predicting corticosteroid resistance and recurrence in keloids: a machine learning study integrating dermatoscopy and ultrasound

**DOI:** 10.3389/fmed.2026.1925984

**Published:** 2026-09-14

**Authors:** Yijia Shao, Jiasheng Hu, Yihui Li, Xianrong Zhu, Jiangyuan Li, Lirong Zheng, Dewei Zhao, Lubin Li, Minmin Lin, Junyi Shao, Zhiming Li

**Affiliations:** 1 Department of Dermatology and Venereology, The First Affiliated Hospital of Wenzhou Medical University, Wenzhou, Zhejiang, China; 2 Wenzhou Medical University, Wenzhou, Zhejiang, China; 3 Department of Urology, Wenzhou Central Hospital, Wenzhou, Zhejiang, China

**Keywords:** clinical prediction model, corticosteroid resistance, dermatoscopy, keloids, machine learning, random forest, recurrence prediction, ultrasound

## Abstract

**Background:**

Keloids are characterized by high recurrence rates and variable responses to intralesional corticosteroid therapy. Objective and non-invasive approaches for predicting treatment response and recurrence remain limited. This study aimed to develop and validate machine learning models integrating dermatoscopic and high-frequency ultrasound features for individualized risk stratification in patients with keloids.

**Methods:**

In this retrospective cohort study, 174 patients with keloids from the internal cohort and an independent external validation cohort of 30 patients were included. Dermatoscopic vascular patterns and ultrasound-derived lesion characteristics were analyzed. Four machine learning algorithms, including Random Forest (RF), Support Vector Machine (SVM), Naive Bayes (NB), and XGBoost, were developed to predict corticosteroid treatment response and recurrence risk. Model performance was evaluated using the area under the receiver operating characteristic curve (AUC), accuracy, sensitivity, specificity, calibration analysis, and decision curve analysis.

**Results:**

For corticosteroid response prediction, the RF model achieved the highest discrimination performance (AUC = 0.816, 95% CI: 0.74–0.88), followed by SVM (AUC = 0.781), NB (AUC = 0.759), and XGBoost (AUC = 0.744). For recurrence prediction, the XGBoost model showed the highest predictive performance (AUC = 0.949, 95% CI: 0.91–0.98). External validation in an independent 30-patient cohort showed comparable discrimination (RF: AUC = 0.851; XGBoost: AUC = 0.855), providing preliminary evidence for model reproducibility. Calibration and decision curve analyses suggested potential clinical usefulness.

**Conclusions:**

Machine learning models integrating dermatoscopic and ultrasound features may provide non-invasive approaches for predicting corticosteroid response and recurrence in keloids. These findings support further multicenter validation before clinical implementation.

## Introduction

Keloid is a benign fibrotic skin disorder characterized by excessive collagen deposition, chronic inflammation, and an invasive growth pattern, often accompanied by neovascularization at the peripheral margins (1, 2). Clinically, it presents as raised, firm scars associated with persistent pruritus or pain (2). In severe cases, scar contractures may impair joint mobility, causing physical and psychological distress to patients. Epidemiological studies indicate that the prevalence of keloids varies significantly across populations, ranging from 1% to 6% worldwide (3). Keloids are highly recurrent and remain a therapeutic challenge, with recurrence rates over 50% following treatment (4). Current treatment options for keloids include intralesional corticosteroid injection, surgical excision, laser therapy, and radiotherapy. Among these, intralesional corticosteroids are considered the first-line treatment due to their anti-inflammatory and antifibrotic effects (5, 6). However, some patients exhibit poor response to corticosteroid therapy, resulting in limited efficacy and early recurrence after treatment cessation. This leads to sustained disease burden with significant psychosocial and economic impacts. Therefore, there is an urgent need to develop predictive methods to support precision therapy.

Emerging evidence suggests that abnormal angiogenesis plays a pivotal role in keloid pathogenesis, treatment response, and recurrence. Recent studies have found that abnormal vascular patterns were observed in keloid lesions (7), and lesions with active angiogenesis tend to respond poorly to corticosteroid therapy (8). Besides, some studies have found that in severe and thick keloids, triamcinolone acetonide is distributed unevenly within the tissue, leading to inconsistent therapeutic outcomes (9). These changes in vascular morphology and lesion thickness may serve as biomarkers for the response to corticosteroid therapy and support predictive models for treatment outcomes.

Dermatoscopy and high-frequency ultrasound have demonstrated significant advantages as non-invasive imaging tools. Dermatoscopy provides high-resolution visualization of vascular morphology (7, 10), while high-frequency ultrasound enables precise measurement of lesion thickness (11). Together, these techniques provide a comprehensive assessment of keloid structures, offering objective imaging features to facilitate non-invasive prediction and support the transition toward personalized therapy.

However, current clinical assessment of keloid sensitivity to corticosteroids largely relies on subjective clinical judgment and retrospective evaluation, lacking objective and standardized criteria. Recent advances in artificial intelligence and machine learning enable the extraction of latent patterns from complex multimodal datasets and facilitate accurate prediction. Therefore, we integrated dermatoscopic and ultrasound features with machine learning algorithms to facilitate the development of predictive models that correlate vascular features with corticosteroid resistance. Such models enable early, non-invasive identification of patients at high risk for treatment resistance and recurrence, supporting timely adjustment of therapeutic strategies. Ultimately, this approach provides important clinical guidance for the personalized diagnosis and treatment of keloids.

## Patients and methods

### Study population and variable selection

This retrospective cohort study enrolled 174 patients with keloids from the Department of Dermatology at the First Affiliated Hospital of Wenzhou Medical University between January 2025 and January 2026. An independent external validation cohort of 30 patients was recruited from Wenzhou Central Hospital using the same eligibility criteria. Adult patients with clinically diagnosed keloids and no prior treatment within the preceding 6 months were included. Patients with malignant tumors, connective tissue diseases, pregnancy or lactation, incomplete clinical records, or loss to follow-up were excluded. Based on treatment outcomes, patients were classified into corticosteroid-sensitive (*n* = 147) and corticosteroid-resistant (*n* = 27) groups. The study protocol was approved by the Institutional Review Board of the First Affiliated Hospital of Wenzhou Medical University (approval number: KY2026-R096) and was conducted in accordance with the Declaration of Helsinki.

### Sample size and machine learning model development

No formal sample size calculation was performed *a priori* because this was an exploratory retrospective study based on consecutive eligible patients. According to the events-per-variable principle proposed by Peduzzi et al. (12), the limited number of corticosteroid-resistant cases (*n* = 27) required strict control of model complexity. Therefore, feature reduction was performed before model development. Candidate predictors were selected based on clinical relevance and univariate associations (*P* < 0.10), followed by recursive feature elimination within the training cohort. The final corticosteroid-response model included five predictors [arborizing vessels, ultrasonographic lesion thickness, Vancouver Scar Scale (VSS), University of North Carolina (UNC) Scar Scale, and linear vessels], while the recurrence model included six predictors (arborizing vessels, lesion thickness, VSS score, lesion length, linear vessels, and UNC score). Feature selection and hyperparameter optimization were conducted exclusively within the training cohort using nested 5-fold cross-validation. Class weighting and bootstrap resampling (1,000 iterations) were applied to improve model stability, and the independent external cohort was used for validation.

### Exclusion of DLQI and HADS from prediction models

Although DLQI and HADS scores were collected as baseline clinical assessments, they were not incorporated into the final machine learning models because the primary objective was to establish an imaging-based predictive framework. These patient-reported measures mainly reflect subjective quality-of-life and psychological status and may be influenced by emotional state and non-lesion-related factors. Therefore, they were considered less specific for intrinsic biological characteristics of keloid lesions compared with dermatoscopic and ultrasonographic features.

### Clinical data collection and outcomes

Clinical data were retrospectively extracted from electronic medical records of patients with keloids managed under standard clinical care. Baseline characteristics were collected, including sex, age, height, weight, course of disease, medication type, and lesion position, size, and origin. All dermatoscopic images, clinical photographs, and high-frequency ultrasound recordings were obtained during standard clinical examinations conducted as part of standard treatment. Patient prognosis was assessed during routine outpatient follow-up visits. The UNC Scar Scale and VSS were used to evaluate symptoms and keloid characteristics respectively, which were independently recorded by two senior dermatologists. Besides, we also recorded the Dermatology life quality index (DLQI) and Hospital anxiety and depression scale (HADS) scores.

Treatment efficacy was defined as a ≥50% reduction in the total VSS score (13) at the 3-month post-treatment evaluation compared with baseline. This cutoff was selected based on previous clinical studies using VSS-based response criteria, in which a ≥50% reduction in VSS score was commonly adopted as a clinically meaningful endpoint reflecting substantial improvement in scar severity and treatment response (13, 14). All treatment response assessments were independently performed by two senior dermatologists who were blinded to baseline dermatoscopic and ultrasound findings and treatment allocation. Inter-rater agreement for VSS assessment was evaluated using the intraclass correlation coefficient (ICC), with an ICC value >0.80 considered indicative of excellent agreement.

Recurrence was defined as a time-to-event outcome, characterized by either an increase in VSS thickness score or the reappearance/worsening of keloid-related symptoms (including pruritus or pain) after completion of active treatment. The recurrence date was defined as the first documented visit fulfilling the recurrence criteria. Patients without recurrence were censored at the date of the last follow-up visit (15, 16). All recurrence assessments were independently reviewed by two dermatologists blinded to the baseline imaging features. For machine learning classification, corticosteroid-sensitive patients were designated as the positive class (1), while corticosteroid-resistant patients were designated as the negative class (0).

Follow-up assessments were conducted at 1, 3, 6, and 12 months after treatment completion, followed by annual evaluations thereafter. The median follow-up duration was 18.6 months (IQR: 12.4–26.8 months; range: 6–36 months). During follow-up, recurrence occurred in 38 of 174 patients (21.8%). The median follow-up duration was 15.2 months (IQR: 10.6–22.5 months) among patients with recurrence and 19.8 months (IQR: 13.6–28.7 months) among patients without recurrence.

### Treatment protocol and corticosteroid exposure

All patients received standardized intralesional corticosteroid-based therapy according to routine clinical practice. Triamcinolone acetonide (40 mg/mL) was the preferred first-line corticosteroid, administered by intralesional injection at a dose of 10–40 mg per session depending on lesion size and thickness. Betamethasone (5 mg/mL) was selected mainly for patients with smaller lesions, superficial lesions, or when a lower injection volume was considered appropriate. The choice between triamcinolone and betamethasone was determined by the treating dermatologist based on lesion characteristics, including thickness, anatomical location, and clinical severity.

Injections were performed at 4-week intervals for a planned treatment course of 3 months (typically 3 sessions). The injection was delivered into the mid-to-deep dermal layer of the keloid lesion using a standardized technique to ensure intralesional deposition. The total number of injections, treatment duration, corticosteroid type, and concomitant medications were recorded for each patient.

For patients with insufficient response or clinically aggressive lesions, concomitant 5-fluorouracil (5-FU, 50 mg/mL) was added at the discretion of the treating dermatologist. The indication for adding 5-FU included poor early response, larger lesions, or higher-risk anatomical sites. The final treatment response was evaluated at 3 months after completion of the planned treatment course.

### Imaging assessment

Dermatoscopy was performed using a digital dermatoscope (CBS-908, China Boshi) under polarized and non-polarized modes (20× magnification). Based on the findings, the vascular structures within the keloids were classified into arborizing, linear, and dotted patterns (7, 17), which were independently evaluated by two experienced dermatologists. Arborizing vessels are characterized by dilated, branched vessels arranged in a network against a structureless background, while Linear vessels appear as discrete, straight vascular structures (18). Dotted vessels manifest as small, red, pinhead-sized dots (Figure 1). High-frequency ultrasound (MD-310S, MEDA) was used to measure lesion thickness (Figure 2). Each lesion was measured three times at the thickest point, and the mean value was recorded.

**Figure 1:**
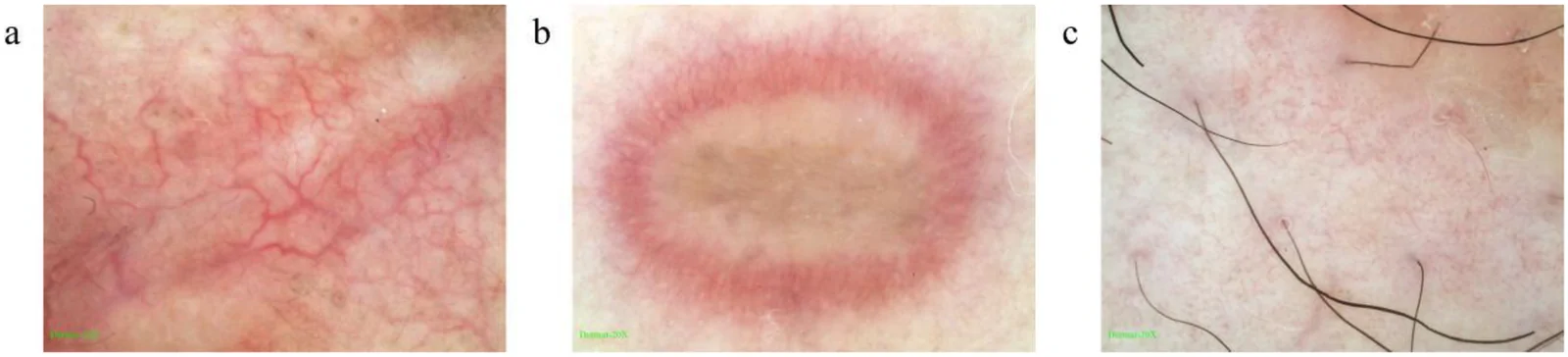
Dermatoscopic vascular patterns in keloids (20× magnification). Representative dermatoscopic images demonstrating the three vascular patterns observed in keloid lesions. **(a)** Arborizing vessels: dilated, irregularly branching vascular structures distributed in a network over a structureless pink-whitish background. **(b)** Linear vessels: discrete, straight, non-branching vascular structures. **(c)** Dotted vessels: small, red, pinhead-sized dots.

**Figure 2:**
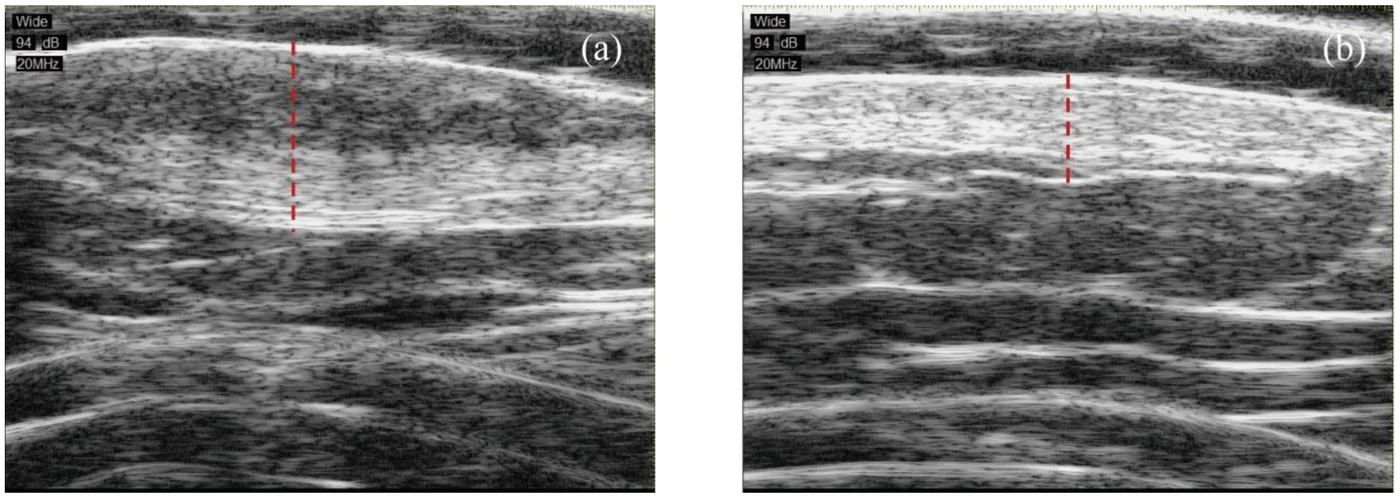
Ultrasound image of patients with keloids (20 MHz). **(a)** Cross-sectional ultrasound image of a keloid lesion. The red dashed line indicates the lesional area. **(b)** Normal skin adjacent to the keloid lesion, serving as an internal reference, with the red dashed line marking the normal dermal layer for comparison.

To assess inter-observer reliability, Cohen's kappa coefficient (*κ*) was calculated for the categorization of vascular patterns between the two evaluators. A *κ* value >0.80 was considered indicative of almost perfect agreement.

### Statistical analysis

Statistical analyses were conducted using SPSS 26.0 (IBM Corp., Armonk, NY, USA) and R software (version 4.3.1, R Foundation for Statistical Computing, Vienna, Austria). Continuous variables were assessed for normality using the Shapiro–Wilk test. Normally distributed data were expressed as mean ± standard deviation (SD) and compared using Student's *t*-test, while non-normally distributed data were presented as median (interquartile range, IQR) and analyzed using the Mann–Whitney *U*-test. Categorical variables were expressed as frequencies and percentages, with comparisons performed using the Chi-square test or Fisher's exact test as appropriate. Subgroup analyses were pre-specified according to sex and age (≤45 years vs. >45 years) to evaluate model stability. All tests were two-tailed, and statistical significance was defined as *p* < 0.05.

Four supervised learning algorithms, including Naive Bayes (NB), Support Vector Machine (SVM), Random Forest (RF), and Extreme Gradient Boosting (XGBoost), were used to develop predictive models for corticosteroid treatment response and recurrence risk. Considering the potential influence of treatment heterogeneity, corticosteroid type (triamcinolone vs. betamethasone) and concomitant 5-FU use were additionally evaluated as treatment-related covariates. Sensitivity analyses were performed by incorporating these treatment exposure variables into the prediction framework to determine whether imaging-derived predictors retained their predictive contribution independent of treatment regimen.

Considering the limited number of corticosteroid-resistant events (*n* = 27), direct inclusion of all candidate variables was avoided. Candidate variables were screened before model construction, and feature selection was conducted only within the training dataset to prevent information leakage. For the corticosteroid-resistance prediction models, five predictors were retained: arborizing vessels, ultrasonographic lesion thickness, VSS score, UNC score, and linear vessels. For recurrence prediction models, six predictors were retained: arborizing vessels, lesion thickness, VSS score, lesion length, linear vessels, and UNC score.

Data were preprocessed using normalization and one-hot encoding. Before model construction, potential multicollinearity among candidate predictors was assessed using variance inflation factors (VIF). All retained predictors showed low collinearity (VIF < 2.5), suggesting that feature correlation was unlikely to substantially influence the ranking of model-derived importance scores. Missing values were minimal (<5%) and were handled using median imputation for continuous variables and mode imputation for categorical variables. Given the class imbalance between corticosteroid-sensitive (*n* = 147) and corticosteroid-resistant (*n* = 27) patients, class weighting was applied in all models to reduce bias toward the majority class.

The internal dataset consisted of 174 patients and was randomly divided into a training cohort (*n* = 122, 70.1%) and an internal testing cohort (*n* = 52, 29.9%) using a 7:3 split ratio. The training cohort was exclusively used for feature selection, preprocessing, and model development, whereas the internal testing cohort was reserved for independent performance evaluation. All preprocessing procedures, including feature selection, normalization, and hyperparameter optimization, were performed within the training cohort only. A nested 5-fold cross-validation strategy was applied for feature selection and model tuning to minimize overfitting and optimism bias. An external validation cohort comprising 30 patients from Wenzhou Central Hospital was subsequently used for independent model evaluation without model retraining. The complete dataset allocation process is illustrated in Figure 3, and cohort assignments are summarized in Supplementary Table S1.

**Figure 3:**
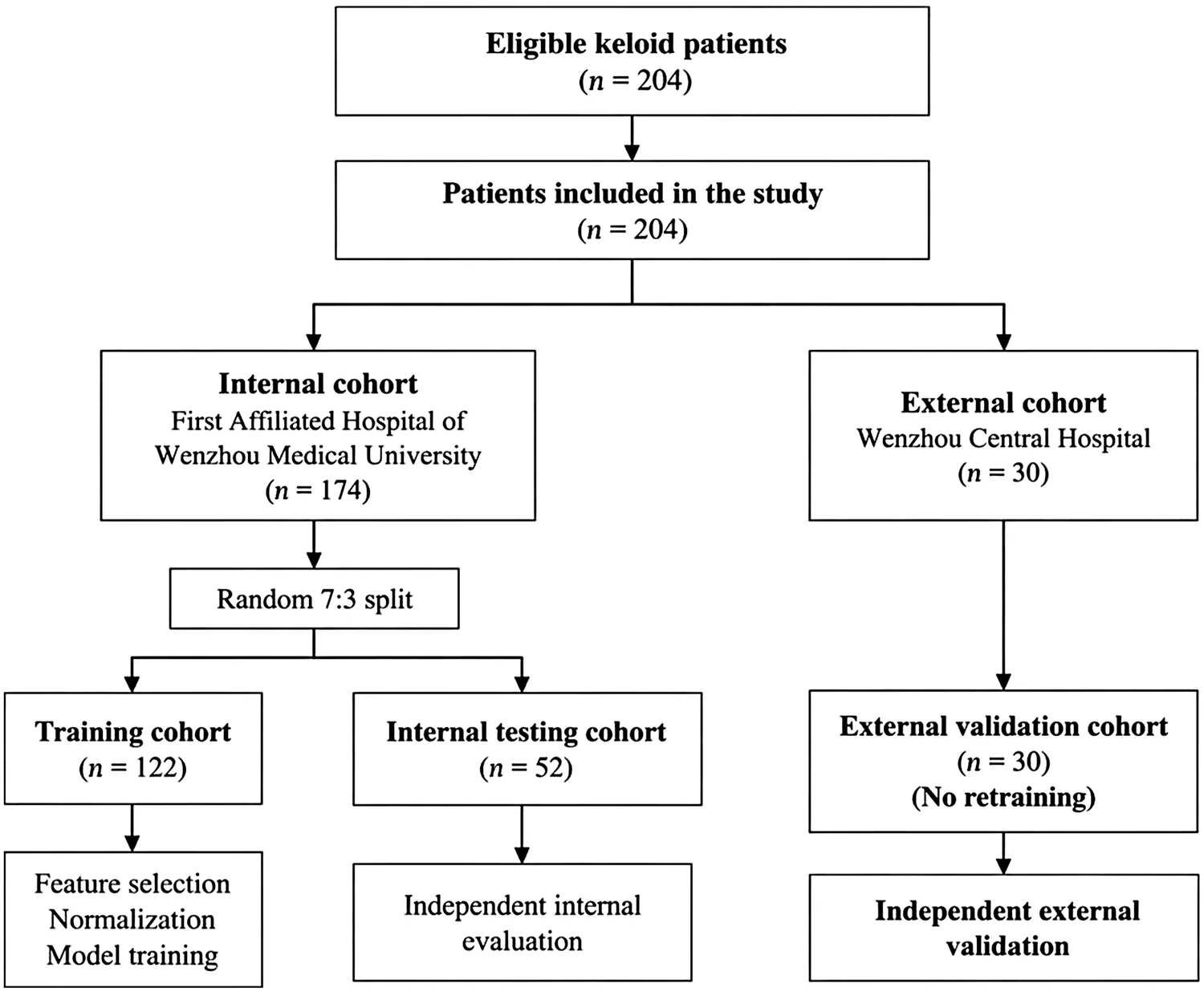
Flowchart of patient selection and dataset allocation.

**Figure 4:**
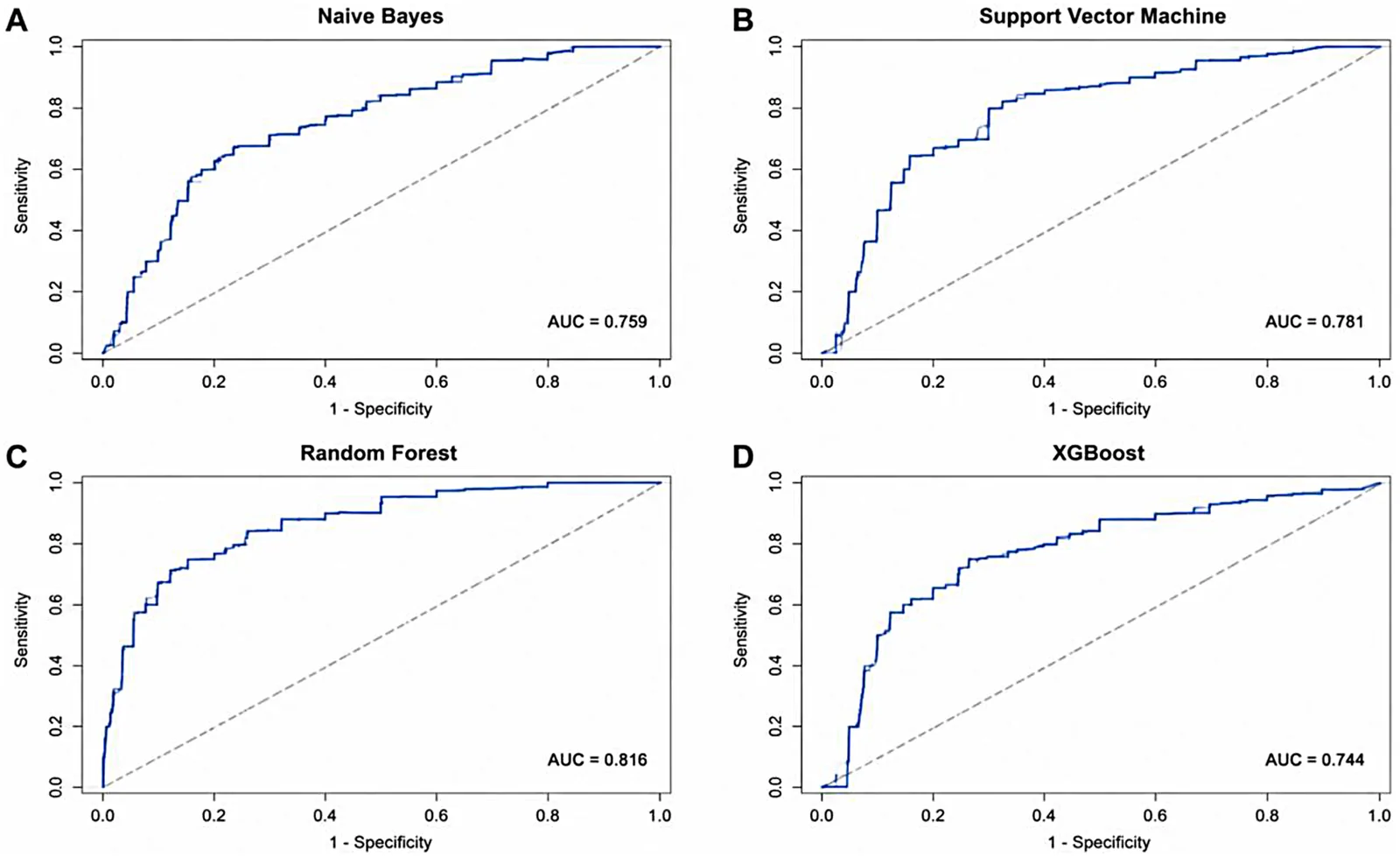
ROC curves of machine learning models for predicting corticosteroid treatment response in keloids. ROC curves and corresponding AUC values are shown for **(A)** Naive Bayes (AUC = 0.759), **(B)** Support Vector Machine (AUC = 0.781), **(C)** Random Forest (AUC = 0.816), and **(D)** XGBoost (AUC = 0.744). The diagonal line represents the reference line (AUC = 0.5), indicating no discriminative ability.

Hyperparameters for each model were optimized using grid search within the nested 5-fold cross-validation framework described above. For Random Forest, the number of trees and maximum depth were tuned; for SVM, the kernel type and regularization parameter (C) were optimized; and for XGBoost, the learning rate, maximum depth, and subsampling ratio were adjusted.

Model discrimination was evaluated using the area under the receiver operating characteristic curve (AUC-ROC), accuracy, sensitivity, specificity, precision, recall, and F1-score. The corresponding 95% confidence intervals (CIs) were estimated using bootstrap resampling. Model calibration was assessed using calibration curves and the Brier score, while the Hosmer–Lemeshow test was used to evaluate agreement between predicted and observed probabilities. Decision curve analysis (DCA) was performed to quantify clinical utility across different threshold probabilities by comparing net benefit with treat-all and treat-none strategies.

To improve model interpretability, permutation importance was calculated for the Random Forest model, and SHapley Additive exPlanations (SHAP) values were generated for the XGBoost model. Feature importance scores were normalized to a 0–1 scale for visualization and comparison, with higher values indicating greater contributions of individual predictors to model predictions.

Because recurrence represents a time-dependent outcome, Kaplan–Meier survival analysis and Cox proportional hazards regression were additionally performed to evaluate recurrence-free survival. Time-dependent ROC analysis was conducted to assess the discriminative ability of recurrence prediction models over different follow-up periods.

All machine learning analyses were conducted using Python version 3.10 with scikit-learn and XGBoost packages, while statistical analyses were performed using R version 4.3.1. A fixed random seed (seed = 42) was applied to ensure reproducibility.

## Results

### Baseline characteristics and univariate analysis

Inter-observer agreement for dermatoscopic vascular pattern classification was excellent, with a Cohen's kappa coefficient of 0.87 (95% CI: 0.80–0.94), indicating high reproducibility of the qualitative vascular assessments. The inter-rater reliability of VSS scoring was also excellent, with an ICC of 0.91 (95% CI: 0.87–0.94), indicating high reproducibility of scar severity assessment between the two blinded dermatologists.

Among the 174 patients included, 79 (45.4%) were male and 95 (54.6%) were female. According to treatment response stratification, 147 patients were corticosteroid-sensitive, whereas 27 patients showed corticosteroid-resistant. Comparative analysis between the corticosteroid-sensitive and corticosteroid-resistant groups revealed that most baseline clinical characteristics were comparable. However, corticosteroid type differed significantly between groups (*P* = 0.007), with a higher proportion of betamethasone use observed in the corticosteroid-resistant group (33.3% vs. 11.6%). Concomitant 5-FU use was comparable between groups (15.0% vs. 11.1%, *P* = 0.770) (Table 1). Importantly, ultrasonographic lesion thickness, VSS scores, UNC scores, and arborizing vessels remained significantly associated with corticosteroid resistance after accounting for treatment-related factors.

**Table 1:** Baseline characteristics of keloid patients stratified by corticosteroid treatment response.

Index	Corticosteroid-sensitive (*n* = 147)	Corticosteroid-resistant (*n* = 27)	*p* value
Sex			0.464
Male	65 (44.2%)	14 (51.9%)	
Female	82 (55.8%)	13 (48.1%)	
Age (years)	40.06 ± 13.99	37.15 ± 10.86	0.306
Height (cm)	166.59 ± 9.61	168.33 ± 10.53	0.395
Weight (kg)	62.37 ± 12.56	65.3 ± 15.81	0.27
Course of disease (years)	2.797 ± 4.74	2.62 ± 3.25	0.858
Corticosteroid type			0.007
Betamethasone	17 (11.6%)	9 (33.3%)	
Triamcinolone	130 (88.4%)	18 (66.7%)	
Concomitant 5-FU use			0.77
Yes	22 (15.0%)	3 (11.1%)	
No	125 (85.0%)	24 (88.9%)	
Lesion position			0.465
Chest	87 (59.2%)	18 (66.7%)	
Non-chest	60 (40.8%)	9 (33.3%)	
Length (cm)	69.01 ± 68.27	91.11 ± 64.35	0.121
Width (cm)	39.95 ± 81.53	53.56 ± 94.41	0.438
Ultrasonographic lesion thickness (mm)	2.89 ± 1.20	4.04 ± 0.90	<0.001
Source			0.675
Spontaneous	88 (59.9%)	15 (55.6%)	
Trauma-induced	59 (40.1%)	12 (44.4%)	
DLQI	2.99 ± 1.80	3.07 ± 1.84	0.831
HADS scores	14.31 ± 5.13	15.07 ± 5.46	0.48
VSS scores	9.79 ± 2.97	12.41 ± 3.61	<0.001
UNC scores	5.97 ± 2.43	7.67 ± 2.06	0.001
Dotted vessels			1
Yes	13 (8.8%)	2 (7.4%)	
No	134 (91.2%)	25 (92.6%)	
Arborizing vessels			<0.001
Yes	9 (6.1%)	14 (51.9%)	
No	138 (93.9%)	13 (48.1%)	
Linear vessels			0.503
Yes	15 (10.2%)	4 (14.8%)	
No	132 (89.8%)	23 (85.2%)	
Median injection sessions	3 (3–4)	3 (2–4)	0.421
Treatment duration (months)	3.0 ± 0.5	3.1 ± 0.6	0.356

DLQI, Dermatology life quality index; HADS, Hospital anxiety and depression scale; VSS, Vancouver Scar Scale; UNC, University of North Carolina Scar Scale.

### Machine learning models for treatment response and recurrence prediction

Four machine learning algorithms, including RF, XGBoost, SVM, and NB, were developed to predict corticosteroid treatment response and recurrence in patients with keloids.

For corticosteroid treatment response prediction, the RF model demonstrated the highest discriminative performance among the four algorithms, achieving an AUC of 0.816 (95% CI: 0.74–0.88) on the internal test set (*n* = 52), with an accuracy of 0.823, sensitivity of 0.808, and specificity of 0.846. The SVM model yielded an AUC of 0.781 (95% CI: 0.71–0.85), with an accuracy of 0.789, sensitivity of 0.775, and specificity of 0.808. The NB model showed an AUC of 0.759 (95% CI: 0.68–0.83), accuracy 0.767, sensitivity 0.750, specificity 0.788. The XGBoost model for treatment response achieved an AUC of 0.744 (95% CI: 0.67–0.82), with accuracy 0.752, sensitivity 0.735, and specificity 0.773 (Figure 4).

For recurrence prediction, the XGBoost model achieved the best discriminative ability, with an AUC of 0.949 (95% CI: 0.91–0.98) on the internal test set, with an accuracy of 0.877, sensitivity of 0.900, and specificity of 0.846.

During follow-up, recurrence occurred in 38 of 174 patients (21.8%), with a median follow-up duration of 18.6 months (IQR: 12.4–26.8 months; range: 6–36 months). Detailed follow-up characteristics and recurrence events are summarized in Supplementary Table S2. For recurrence prediction, the XGBoost model achieved the best discriminative ability, with an AUC of 0.949 (95% CI: 0.91–0.98), followed by RF (AUC = 0.929), SVM (AUC = 0.913), and NB (AUC = 0.788) (Figure 5). The recurrence prediction model was constructed using only pretreatment variables, including baseline dermatoscopic vascular patterns, ultrasonographic lesion thickness, VSS score, lesion length, linear vessels, and UNC score, without incorporating any post-treatment information.

**Figure 5:**
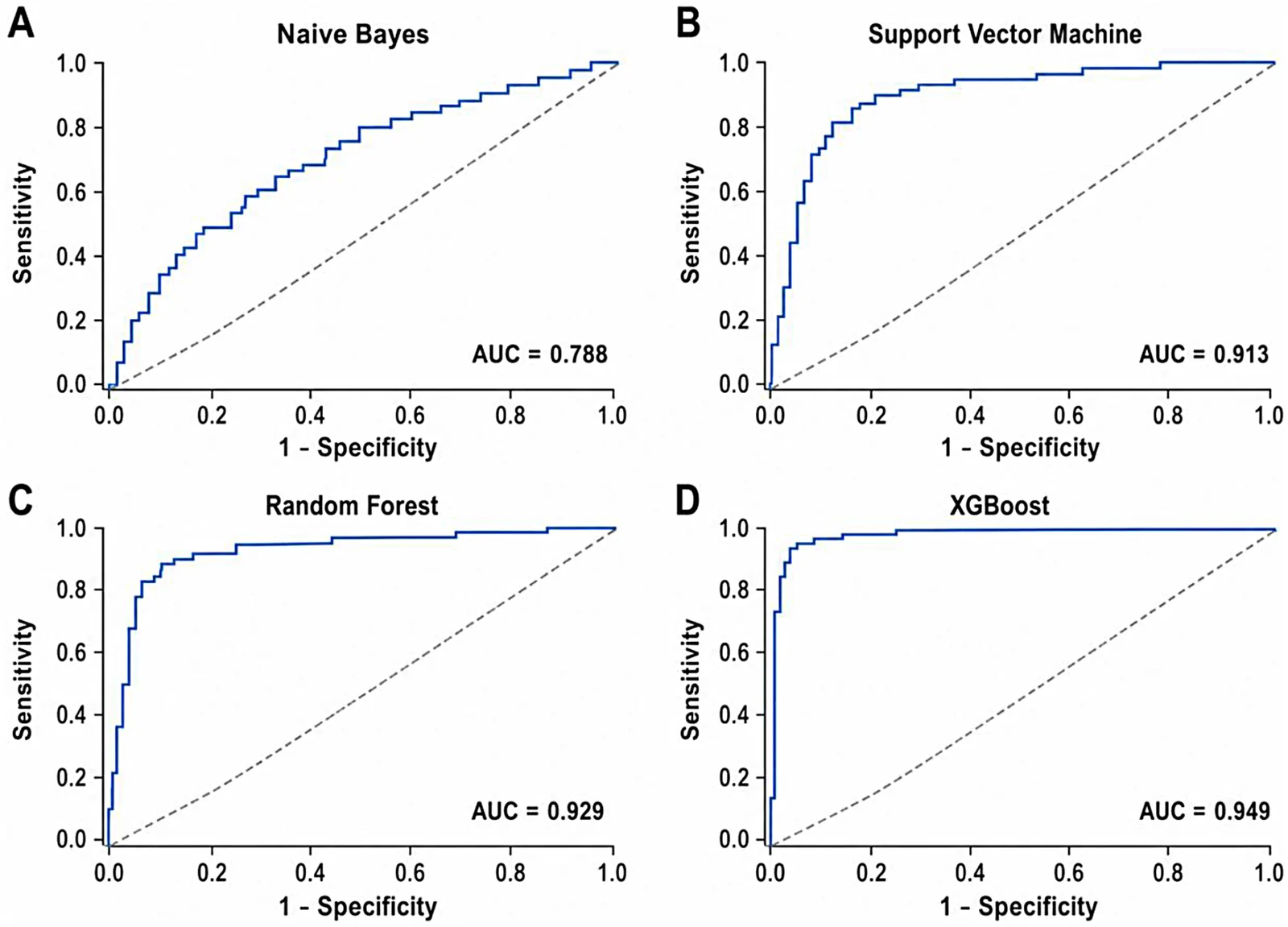
ROC curves of machine learning models for predicting keloid recurrence. ROC curves and corresponding AUC values are shown for **(A)** Naive Bayes (AUC = 0.788), **(B)** Support Vector Machine (AUC = 0.913), **(C)** Random Forest (AUC = 0.929), and **(D)** XGBoost (AUC = 0.949). The diagonal line represents the reference line (AUC = 0.5), indicating no discriminative ability.

Feature importance analysis demonstrated that vascular morphology and lesion thickness were key contributors to model prediction. In the RF treatment-response model, normalized permutation importance identified arborizing vessels as the most influential predictor (0.41), followed by ultrasonographic lesion thickness (0.22), VSS score (0.19), UNC score (0.12), and linear vessels (0.06).

For recurrence prediction, SHAP analysis of the XGBoost model showed a similar contribution pattern. Arborizing vessels exhibited the highest mean absolute SHAP value (0.38), followed by lesion thickness (0.24), VSS score (0.18), lesion length (0.12), linear vessels (0.05), and UNC score (0.03). Although the absolute values derived from permutation importance and SHAP analysis represent different model-specific interpretation metrics, both approaches consistently identified arborizing vessels and lesion thickness as major predictors across algorithms (Figure 6).

**Figure 6:**
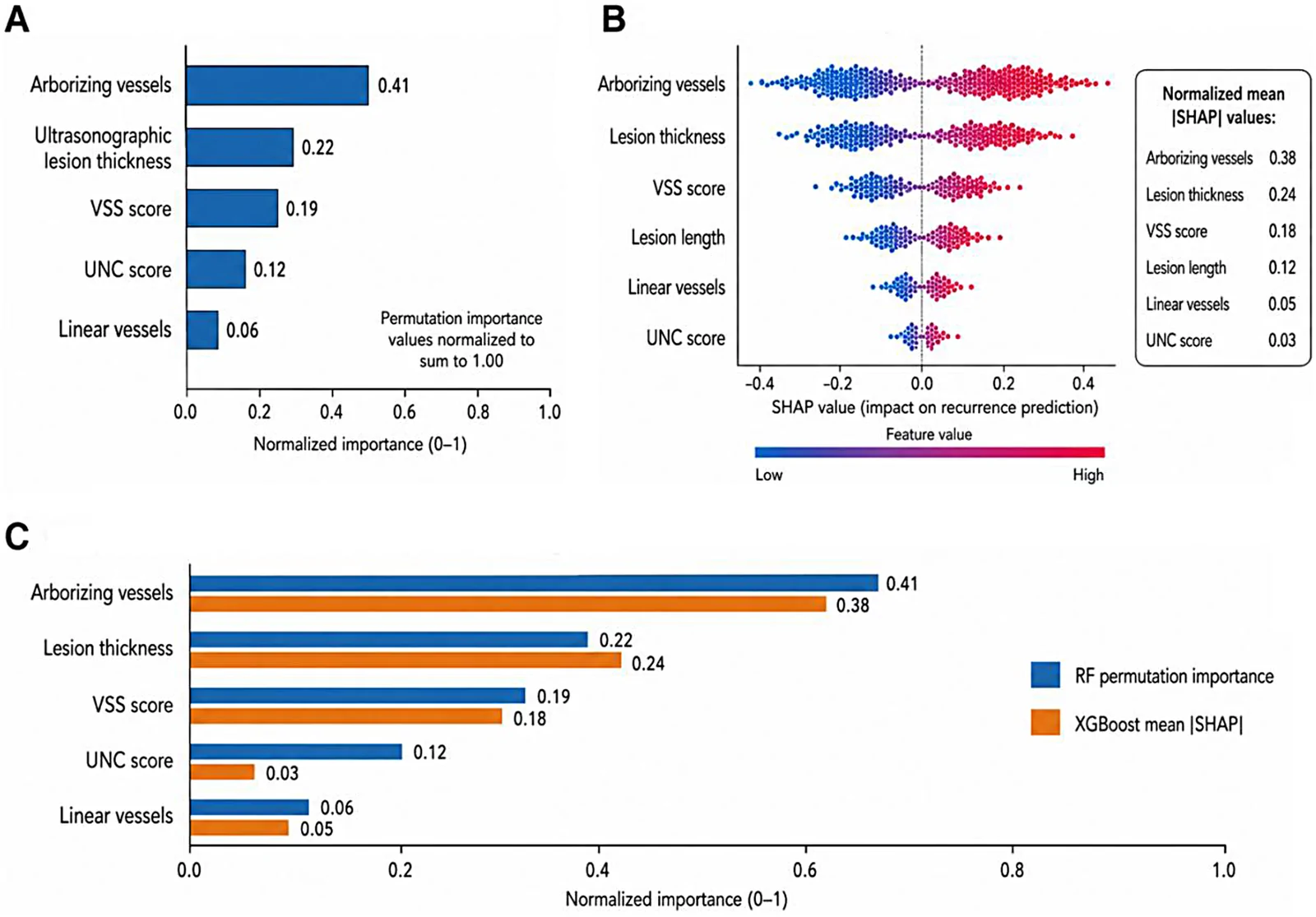
Model-specific feature importance analysis and SHAP interpretation of machine learning predictors. **(A)** Normalized permutation importance ranking of predictors in the Random Forest model for corticosteroid treatment response prediction. Arborizing vessels showed the greatest contribution, followed by ultrasonographic lesion thickness, VSS score, UNC score, and linear vessels. **(B)** SHAP summary plot illustrating the contribution of individual predictors in the XGBoost recurrence prediction model. Arborizing vessels and lesion thickness demonstrated the largest mean absolute SHAP values. **(C)** Comparison of predictor contribution patterns between Random Forest permutation importance and XGBoost SHAP interpretation. Both approaches consistently identified vascular morphology and lesion thickness as major contributors to model predictions.

### Sensitivity analysis accounting for treatment exposure

To evaluate whether treatment heterogeneity influenced model performance, sensitivity analyses were performed by incorporating corticosteroid type and concomitant 5-FU use as additional treatment-related covariates (Supplementary Table S3). After adjustment for treatment regimen, the RF model maintained comparable discrimination, with an AUC of 0.803 (95% CI: 0.73–0.87), compared with the primary model (AUC = 0.816, 95% CI: 0.74–0.88). Furthermore, a subgroup analysis restricted to patients receiving triamcinolone monotherapy demonstrated similar predictive performance (AUC = 0.809, 95% CI: 0.72–0.88), supporting the stability of the model independent of corticosteroid regimen (Supplementary Table S4). Arborizing vessels and ultrasonographic lesion thickness remained the most influential predictors after accounting for treatment exposure, suggesting that the predictive performance was mainly driven by intrinsic lesion characteristics rather than treatment selection alone.

### External validation

In the external validation cohort (*n* = 30), the RF treatment-response model achieved an AUC of 0.851 (95% CI: 0.73–0.95), with an accuracy of 0.833, sensitivity of 0.818, and specificity of 0.846. For recurrence prediction, the XGBoost model achieved an AUC of 0.855 (95% CI: 0.74–0.96), with an accuracy of 0.867, sensitivity of 0.889, and specificity of 0.833 (Table 2). Given the limited sample size of the external validation cohort, these findings should be interpreted as preliminary external validation rather than definitive evidence of broad generalizability.

**Table 2:** External validation and calibration performance of ML models.

Model	AUC (95% CI)	Accuracy	Sensitivity	Specificity
RF (treatment response)	0.851 (0.73–0.95)	0.833	0.818	0.846
XGBoost (recurrence)	0.855 (0.74–0.96)	0.867	0.889	0.833

Calibration analysis demonstrated good agreement between predicted and observed outcomes. The Random Forest model showed a Brier score of 0.118, and the Hosmer–Lemeshow test indicated no significant deviation (*χ*^2^ = 6.42, *p* = 0.601), suggesting good model calibration.

### Time-to-event analysis of recurrence

During a median follow-up of 18.6 months, recurrence-free survival differed significantly between XGBoost-predicted risk groups. Kaplan–Meier analysis demonstrated that patients classified as high-risk had significantly shorter recurrence-free survival than those classified as low-risk (log-rank *P* < 0.001; Figure 7).

**Figure 7:**
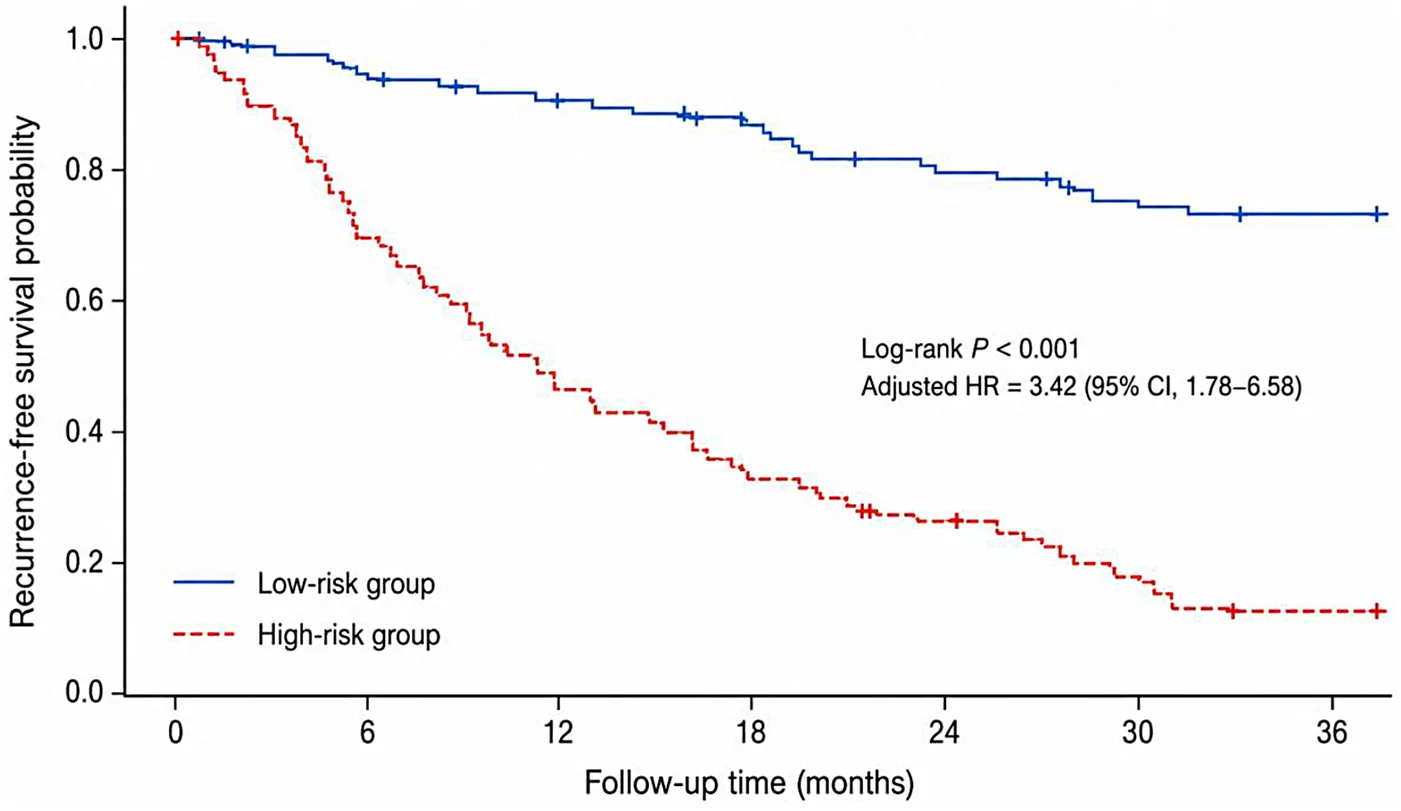
Kaplan–meier analysis of recurrence-free survival stratified by XGBoost-predicted recurrence risk.

In Cox proportional hazards regression analysis, the XGBoost-derived high-risk classification remained independently associated with increased recurrence risk after adjustment for age, sex, lesion location, and disease duration (HR = 3.42, 95% CI: 1.78–6.58, *P* < 0.001; Supplementary Table S5). These findings further supported the clinical relevance and temporal stability of the recurrence prediction model.

Kaplan–Meier curves showing recurrence-free survival according to XGBoost-predicted recurrence risk categories. Patients classified as high-risk demonstrated significantly shorter recurrence-free survival compared with low-risk patients (log-rank *P* < 0.001).

### Subgroup analysis

Subgroup analyses demonstrated consistent performance of the RF model across sex and age strata, with AUCs ranging from 0.789 to 0.912 (Table 3), indicating good model stability across different clinical subgroups.

**Table 3:** Subgroup analysis of RF model performance.

Subgroup	AUC	95% CI	Accuracy	Sensitivity	Specificity
Male	0.789	0.70–0.86	0.81	0.78	0.82
Female	0.84	0.77–0.90	0.83	0.8	0.85
≤45 years	0.867	0.80–0.93	0.85	0.83	0.86
>45 years	0.912	0.85–0.96	0.88	0.86	0.90

Decision curve analysis showed that both the Random Forest and XGBoost models achieved higher net clinical benefit across threshold probabilities of 0.1–0.6 compared with treat-all or treat-none strategies, supporting their potential clinical utility for decision-making.

Bootstrap validation (1,000 resamples) further confirmed model robustness, with minimal variation in AUC (±0.02) across resampling iterations.

## Discussion

In this study, we combined dermatoscopic vascular features with machine learning algorithms to develop predictive models for corticosteroid treatment response and recurrence in keloids. Among 174 patients, conventional clinical variables including sex, age, disease duration, lesion location, and size showed limited predictive value for identifying corticosteroid resistant keloids. This finding suggests that traditional assessments based on macroscopic appearance are poor predictors of treatment outcomes. In contrast, dermatoscopic vascular patterns, particularly arborizing vessels, were significantly associated with corticosteroid resistance. Among the evaluated algorithms, Random Forest performed best for predicting treatment response, whereas XGBoost demonstrated superior performance for recurrence prediction. External validation in an independent cohort yielded consistent results, though the sample size was small and derived from the same geographic region, which may limit the assessment of broader generalizability.

Feature interpretation using normalized importance metrics consistently identified arborizing vessels as the most influential predictor across models. In the Random Forest resistance model, arborizing vessels showed the highest normalized permutation importance of 0.41, followed by ultrasonographic lesion thickness at 0.22, VSS score at 0.19, UNC score at 0.12, and linear vessels at 0.06. Similarly, SHAP analysis of the XGBoost recurrence model revealed a comparable ranking pattern, with arborizing vessels having the largest mean absolute SHAP value of 0.38. Ultrasound derived lesion thickness also showed a significant association with corticosteroid resistance in univariate analysis; however, within the machine learning framework, arborizing vessels exhibited a stronger correlation with treatment resistance. Subgroup analyses demonstrated stable predictive performance across sex and age strata, with relatively higher accuracy observed in patients older than 45 years. Although some imaging features may be biologically interrelated, formal multicollinearity assessment confirmed low correlation among retained predictors with all variance inflation factors below 2.5, suggesting that the observed importance rankings were unlikely to be substantially distorted by redundant variables.

The higher frequency of arborizing vessels observed in the corticosteroid resistant group compared with the sensitive group suggests that abnormal vascular morphology may be associated with reduced treatment responsiveness. Previous studies have shown that aberrant angiogenesis in keloids correlates with fibroblast activation, extracellular matrix remodeling, and disease progression (19–21). These biological alterations may contribute to impaired response to corticosteroid therapy. Among dermatoscopic vascular patterns, arborizing vessels may reflect active vascular remodeling within keloid lesions (10). Beyond vascular density, this specific morphology indicates immature and dysfunctional vascular remodeling, which is closely linked to endothelial mesenchymal transition and chronic inflammatory infiltration (22). The therapeutic efficacy of intralesional corticosteroids depends largely on their capacity to suppress inflammation and inhibit angiogenesis. Therefore, prominent arborizing vessels may represent a more active lesion phenotype, which could partially explain reduced corticosteroid responsiveness (23, 24). In addition, thicker keloids contain a higher density of disorganized vascular structures and extracellular matrix, which may impede drug diffusion and retention, thereby limiting local bioavailability (25–27). Collectively, these findings support an association between arborizing vessels and corticosteroid response patterns and suggest that this feature may contribute meaningfully to machine learning based risk stratification.

Currently, the evaluation of keloid sensitivity to corticosteroid therapy relies predominantly on clinical experience without objective assessment tools. Consequently, patients who are resistant to corticosteroids may undergo prolonged ineffective monotherapy, delaying timely initiation of combination strategies. As a widely available and cost effective imaging modality, dermatoscopy offers advantages including non invasiveness, ease of performance, and high reproducibility. The predictive model developed in this study enables non invasive pretreatment risk stratification for patients with keloids. Those identified as high risk for corticosteroid resistance could benefit from early treatment adjustments, such as reducing reliance on repeated corticosteroid injections and incorporating combination therapies. Similarly, identifying patients at elevated risk of recurrence facilitates the design of personalized follow up schedules. Compared with traditional approaches that depend on histopathology or single clinical indicators, dermatoscopy allows direct *in vivo* observation of microvascular morphology, making it more feasible in routine clinical practice and even primary care settings. Arborizing vessels showed potential value as an imaging feature associated with both treatment response and recurrence risk, supporting further investigation of vascular characteristics in keloid risk stratification.

Several limitations should be acknowledged. First, although we performed external validation, this was a single center retrospective study and therefore cannot exclude potential selection bias. Moreover, the external validation cohort was recruited from a hospital within the same city, which may introduce similarities in clinical practice patterns and limit the assessment of true cross population generalizability. Second, the dermatoscopic assessment of vascular morphology remains partially subjective. Future studies incorporating automated vessel segmentation and quantitative image analysis may help improve objectivity and reproducibility. Third, this study focused exclusively on objective dermatoscopic and high frequency ultrasound features. Although patient reported measures including DLQI and HADS were collected, these variables were not incorporated into the predictive models because they primarily reflect subjective quality of life and psychological dimensions rather than lesion specific biological characteristics. Future research may explore whether combining imaging biomarkers with validated patient reported outcomes further enhances predictive performance. Fourth, treatment heterogeneity represents an additional limitation. Although intralesional corticosteroid therapy followed a relatively consistent clinical protocol, the use of different corticosteroid preparations and occasional concomitant 5 FU administration reflected real world practice variation. Sensitivity analyses incorporating treatment related variables demonstrated that the predictive contribution of dermatoscopic and ultrasound features remained stable. Nevertheless, corticosteroid resistance should be interpreted as resistance to the applied treatment regimen rather than an absolute biological resistance phenotype. Future prospective studies with standardized corticosteroid protocols and predefined dosing schedules are warranted. Therefore, the reported model performance should be viewed as exploratory rather than definitive, and further multicenter investigations with larger sample sizes are required before clinical implementation. Fifth, although an external validation cohort was included, its limited size restricted the precision of performance estimates and may have constrained the assessment of model generalizability. Accordingly, this external validation should be considered a proof of concept evaluation rather than definitive confirmation of clinical applicability. Future multicenter studies involving larger and more diverse populations are necessary to further validate model performance and establish generalizability across different clinical settings. Finally, although recurrence was evaluated using time to event analyses in addition to binary classification models, the retrospective design and variable follow up duration may have introduced residual bias related to follow up intensity and censoring patterns. Prospective multicenter studies with predefined follow up schedules are needed to further validate the recurrence prediction model. Incorporation of multi omics data may further enhance predictive performance in future investigations.

In conclusion, we developed machine learning models based on dermatoscopic vascular features to predict corticosteroid resistance and recurrence in patients with keloids. With arborizing vessels identified as a key predictor, these models may serve as potential non invasive tools for predicting treatment outcomes, pending further validation in larger multicenter cohorts. If confirmed, they could provide clinicians with evidence based guidance for pretreatment risk stratification and personalized management of keloids.

## Data Availability

The raw data supporting the conclusions of this article will be made available by the authors, without undue reservation.
