# Supplementary material for: Predicting corticosteroid resistance and recurrence in keloids: a machine learning study integrating dermatoscopy and ultrasound
Source: Front Med (Lausanne). 2026 Sep 14;13:1925984. doi: 10.3389/fmed.2026.1925984 (PMC13616790; doi:10.3389/fmed.2026.1925984)
Supplement: Supplementary file 1 [file Supplementaryfile1.docx]

**Predicting Corticosteroid Resistance and Recurrence in Keloids: A Machine Learning Study Integrating Dermatoscopy and Ultrasound**

**Supplementary Table 1. Dataset allocation and model evaluation cohorts**

| **Cohort** | **Purpose** | **Sample size** | **Model evaluation** |
| --- | --- | --- | --- |
| Internal training cohort | Model development, feature selection, normalization, and hyperparameter optimization | n = 122 | RF, SVM, NB, and XGBoost model training |
| Internal testing cohort | Independent internal performance evaluation after model development | n = 52 | RF, SVM, NB, and XGBoost model testing |
| External validation cohort | Independent external validation without model retraining | n = 30 | RF treatment-response model and XGBoost recurrence model evaluation |

**Supplementary Table 2. Follow-up characteristics and recurrence events in the study cohort**

| **Variable** | **Total cohort (n=174)** |
| --- | --- |
| Median follow-up duration, months | 18.6 (12.4–26.8) |
| Follow-up range, months | 6–36 |
| Recurrence events, n (%) | 38 (21.8%) |
| No recurrence, n (%) | 136 (78.2%) |
| Median follow-up among recurrence patients, months | 15.2 (10.6–22.5) |
| Median follow-up among non-recurrence patients, months | 19.8 (13.6–28.7) |

**Supplementary Table 3. Sensitivity analysis incorporating treatment exposure variables into the Random Forest model for predicting corticosteroid resistance**

| **Model** | **Included predictors** | **Treatment-related covariates** | **AUC (95% CI)** | **Sensitivity** | **Specificity** |
| --- | --- | --- | --- | --- | --- |
| Primary RF model | Arborizing vessels, ultrasonographic lesion thickness, VSS score, UNC score, linear vessels | None | 0.816 (0.74–0.88) | 91.50% | 80.00% |
| Adjusted RF model | Arborizing vessels, ultrasonographic lesion thickness, VSS score, UNC score, linear vessels | Corticosteroid type + concomitant 5-FU use | 0.803 (0.73–0.87) | 89.80% | 78.60% |

Abbreviations: RF, Random Forest; VSS, Vancouver Scar Scale; UNC, University of North Carolina Scar Scale; 5-FU, 5-fluorouracil.

**Supplementary Table 4. Performance of the Random Forest model restricted to patients receiving triamcinolone monotherapy**

| **Model** | **Population** | **AUC (95% CI)** |
| --- | --- | --- |
| RF model | All patients (n=174) | 0.816 (0.74–0.88) |
| RF model | Triamcinolone monotherapy subgroup (n=148) | 0.809 (0.72–0.88) |

**Supplementary Table 5. Cox proportional hazards regression analysis for recurrence-free survival**

| **Variable** | **HR** | **95% CI** | **P value** |
| --- | --- | --- | --- |
| XGBoost-predicted high-risk group | 3.42 | 1.78–6.58 | <0.001 |
| Age (per year) | 1.02 | 0.99–1.05 | 0.21 |
| Male sex | 1.18 | 0.63–2.21 | 0.61 |
| Chest lesion location | 1.31 | 0.71–2.42 | 0.39 |
| Disease duration (per year) | 1.04 | 0.96–1.13 | 0.34 |
